# Effectiveness of E‐Learning in Undergraduate ENT Education: A Mixed‐Methods Systematic Review

**DOI:** 10.1002/lary.70164

**Published:** 2025-09-27

**Authors:** Zahir Mughal, Keshav Kumar Gupta, Rosalind di Traglia, Birgit Fruhstorfer

**Affiliations:** ^1^ Department of ENT John Radcliffe Hospital Oxford UK; ^2^ Department of Medical Education University of Warwick Medical School, University of Warwick Coventry UK; ^3^ Department of ENT Worcestershire Royal Hospital Worcester UK; ^4^ Department of ENT New Cross Hospital Wolverhampton UK

**Keywords:** education, internet, learning, otolaryngology, technology

## Abstract

**Objective:**

Ear, nose and throat (ENT) is often underrepresented in undergraduate medical curricula. E‐learning has emerged as a promising strategy to address this gap. This mixed‐methods systematic review aimed to evaluate the effectiveness of e‐learning in undergraduate ENT education.

**Data Sources:**

MEDLINE, Embase, Education Research Complete, and Web of Science were searched.

**Review Methods:**

The PRISMA guidelines were followed. Quantitative data were synthesized with a meta‐analysis of normalized gain scores to assess knowledge improvement. Qualitative findings were analyzed using thematic synthesis. Due to substantial heterogeneity, a narrative synthesis was performed for skills and confidence.

**Results:**

Twenty‐nine studies fulfilled the inclusion criteria. Knowledge scores improved by 26.8% (95% CI 23.7%–30.0%, *n* = 13), which was considered low gain. Skill improvement was variable (*n* = 6), while confidence consistently improved (*n* = 5). Thematic synthesis identified four themes: (1) multimedia resources, (2) learner autonomy and self‐directed engagement, (3) technical barriers, and (4) blended learning models.

**Conclusion:**

Knowledge and learner confidence improved with e‐learning. However, its impact on practical skills was limited. These findings support the integration of e‐learning as a complementary adjunct to clinical teaching.

## Graphical abstract

E‐learning in undergraduate ear, nose, and throat education was evaluated in a mixed‐methods systematic review of 29 studies. Pooled analysis showed a modest knowledge gain of 26.8%, variable effects on skills, and consistent improvements in learner confidence. These findings support e‐learning as a useful adjunct, rather than a standalone replacement, for clinical teaching.

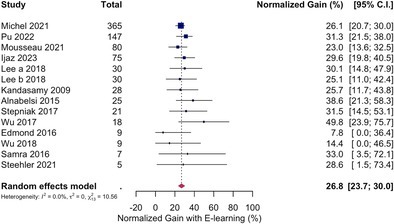

## 1 Introduction

Exposure to ear, nose and throat (ENT) at medical school varies widely across the globe. In the United States (US), only 34% of medical schools provide a formal ENT placement, compared to 78% in the United Kingdom (UK) [1]. Placement durations also vary significantly, ranging from 4 h to 4 months [1]. This inconsistency contributes to frequent reports of low confidence among medical students and resident doctors in the assessment and management of ENT conditions [2, 3]. Students and resident doctors feel this educational gap is best addressed at the undergraduate level [3, 4].

Considerable inconsistencies in ENT curricular content and teaching methods persist across UK medical schools [5, 6]. The recent introduction of the UK Medical Licensing Assessment (MLA)—a national examination for final year medical students [7], has created a need to address disparities between medical schools.

E‐learning has emerged as promising tool to address these challenges. Its advantages include delivery of standardized, accessible, and scalable educational content [8]. The Coronavirus Disease 2019 (COVID‐19) pandemic accelerated the adoption of e‐learning in medical education [9]. Consequently, nearly two‐thirds of UK medical schools incorporate online components into their ENT teaching [10]. Despite its growing presence, the impact of e‐learning on educational outcomes in ENT remains under investigated.

This mixed‐methods systematic review aims to evaluate the effectiveness of e‐learning in undergraduate ENT education. By synthesizing the current evidence, this review seeks to clarify the role of e‐learning in ENT curricula and inform future curricular design.

## 2 Methods

### 2.1 Study Design

This review was reported in accordance with the Preferred Reporting Items for Systematic Review and Meta‐Analysis (PRISMA) guidelines [11]. The review protocol was pre‐registered with the Open Science Framework (https://doi.org/10.17605/OSF.IO/8QF7D). The overarching research question was: *How effective is e‐learning in improving knowledge, skills*, *and confidence among medical students in ENT?* This was divided into two sub‐questions: (1) Quantitative: *What is the magnitude of improvement in these outcomes?* (2) Qualitative: *What are medical students' perceptions and experiences of e‐learning that may explain the observed quantitative findings?*

### 2.2 Search Strategy

A systematic search of the following electronic databases was performed initially on February 26, 2024 and updated on 17 July 2025: Ovid MEDLINE, Ovid Embase, EBSCO Education Research Complete, and Web of Science Core Collection (see Appendix S1). Additional studies were identified through forward citation tracking and reference lists. The search was limited to English‐language articles published within the last 15 years (from January 1, 2010 to date of search). This timeframe was selected to ensure that the included studies reflected modern e‐learning practices rather than outdated technologies, while still providing a sufficiently long timeframe to capture a comprehensive body of literature.

### 2.3 Eligibility Criteria

Inclusion and exclusion criteria were developed using the PICO framework [12], and are summarized in Table 1. Studies were eligible if they involved undergraduate medical students using e‐learning for ENT education and reported quantitative outcomes (knowledge, skills, confidence) or qualitative insights (perceptions, experiences). For the purposes of this review, ‘undergraduate’ refers to students undertaking primary medical training leading to their first medical qualification, irrespective of whether this occurs at the bachelor's or graduate‐entry level.

**TABLE 1:** Inclusion and exclusion criteria.

	Inclusion	Exclusion
Population	Medical students	Other healthcare students or postgraduates
Intervention	E‐learning intervention, defined as teaching and learning via digital technologies, specifically relating to ENT	Other teaching methods such as simulation and virtual reality
Comparison	Not applicable	Not applicable
Outcome	The quantitative outcomes were knowledge, skills, and confidence. The qualitative outcomes were experiences and perceptions	Outcomes related to operative skills, topics beyond the scope of undergraduate ENT education, and career choices
Study design	Observational and experimental study designs	Course descriptions, validation studies, conference abstracts, and review articles

### 2.4 Data Extraction

All retrieved records were imported and de‐duplicated in EndNote Desktop [13]. Two reviewers independently screened titles and abstracts using Rayyan.ai [14]. Full‐text screening was performed independently against the eligibility criteria. Disagreements were resolved through discussion with a third reviewer.

Data were extracted into a pre‐designed table, including the following fields: title, author, year of publication, study design, country, sample size, e‐learning intervention details, participant characteristics, and reported outcomes. Data were extracted independently by one reviewer and verified by a second reviewer for accuracy.

### 2.5 Quality Assessment

Methodological quality was assessed using the Mixed Methods Appraisal Tool (MMAT) [15]. The MMAT assesses studies based on five criteria tailored to their respective study designs. Two reviewers conducted the appraisal independently, and disagreements were resolved through discussion. In addition, each study's educational impact was mapped to Kirkpatrick's model of training evaluation, categorizing outcomes from learner reactions to behavior change [16].

### 2.6 Data Analysis

**FIGURE 1:**
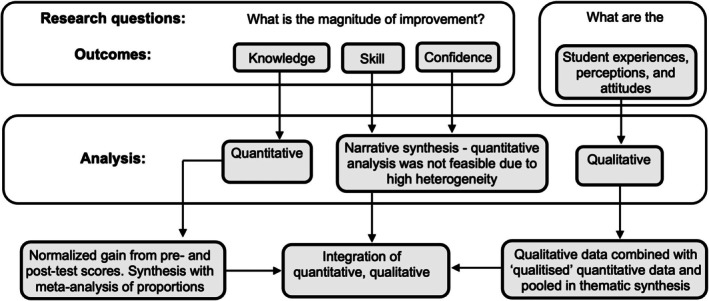
Convergent segregated approach to data synthesis and integration in mixed‐methods review, based on Stern et al. [17]

Quantitative and qualitative data were analyzed using a ‘convergent segregated’ approach in accordance with guidance from the Joanna Briggs Institute (JBI) Mixed Methods Methodology Group [17], as outlined in Figure 1.

#### 2.6.1 Primary Outcome: Knowledge

Knowledge improvement was assessed using pre‐ and post‐intervention scores. The effect size was calculated using normalized gain, a standardized metric described by Hake [18]. This measure reflects how much participants' scores increase relative to the maximum amount. The result ranges from 0 to 1, with values of ≥ 0.7 indicating high gain, 0.3–0.7 medium gain, and < 0.3 low gain [18]. Normalized gain was converted to a percentage to aid interpretation.

To estimate the pooled normalized gain, a meta‐analysis of proportions was conducted using transformed data via the Freeman & Tukey method [19]. A random‐effects model was applied using the DerSimonian & Laird method for estimating between‐study variance (2) [20]. Heterogeneity was further assessed using the *I* ^2^ statistic and the Cochran *Q*‐test. Sensitivity analysis was conducted using leave‐one‐out (LOO) analysis to identify potential outliers.

Subgroup analysis was conducted using a mixed‐effects model, stratifying studies by study design—randomized controlled trials (RCT) versus pre‐post studies; stage of training—early years (first 3 years) versus advanced medical students; and timing of delivery—synchronous versus asynchronous. Meta‐regression was used to explore moderator effects, such as the impact of repeat testing and time allocated to e‐learning. The *R* ^2^ index quantified the proportion of heterogeneity that could be explained by the moderator.

Publication bias was assessed through funnel plot inspection and the unweighted Egger's test. All analyses were performed in *R* version 4.4.2 (The R Foundation for Statistical Computing, Vienna, Austria) [21], using *metafor* and *meta* packages [22, 23]. Findings were reported with 95% confidence intervals (C.I.), and the significance level was < 5%.

#### 2.6.2 Secondary Outcomes: Skills, Confidence, and Learner Experience

The analytical approach to secondary outcomes is summarized in Figure 1. Due to significant heterogeneity in outcome measures and reporting formats for skill and confidence outcomes, meta‐analysis of the quantitative data for these two domains was not feasible. These outcomes were therefore analyzed in a narrative synthesis in the qualitative findings section.

Learner experiences and perceptions were analyzed qualitatively using thematic synthesis. This was performed in a three‐step process: [24] (1) line‐by‐line coding of qualitative data, (2) development of descriptive themes from grouped codes, and (3) generation of higher‐order analytical themes to identify overarching factors influencing the effectiveness of e‐learning.

Learner experiences and perceptions data were available in both qualitative and quantitative formats. While quantitative data would be typically excluded from qualitative synthesis under a *convergent segregated* approach [17], doing so here would have led to loss of valuable contextual insight. Therefore, a *qualitization* process was undertaken, whereby numerical findings were transformed into descriptive summaries [17].

## 3 Results

### 3.1 Study Selection

**FIGURE 2:**
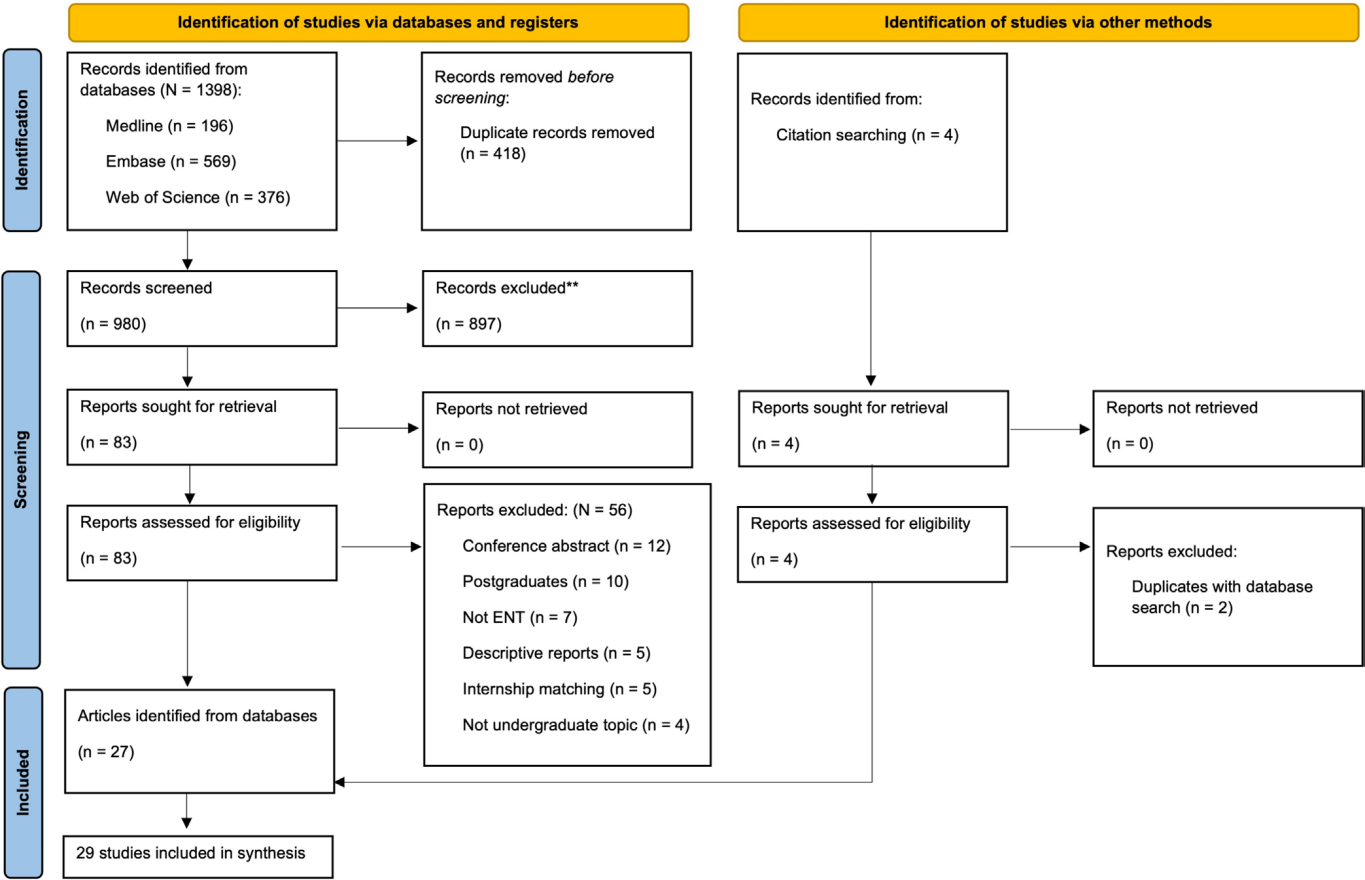
PRISMA flow diagram. [Color figure can be viewed in the online issue, which is available at www.laryngoscope.com]

A total of 1398 records were identified from MEDLINE (*n* = 196), Embase (*n* = 569), Web of Science (*n* = 376), and Education Research Complete (*n* = 257). An additional four records were identified through citation tracking and reference lists. After screening, 29 studies met the eligibility criteria and were included in the final analysis [8, 25, 26, 27, 28, 29, 30, 31, 32, 33, 34, 35, 36, 37, 38, 39, 40, 41, 42, 43, 44, 45, 46, 47, 48, 49, 50, 51, 52]. Reasons for exclusion are detailed in the PRISMA flow diagram—see Figure 2.

### 3.2 Study Characteristics

Study and participant characteristics are summarized in Tables 2 and 3, respectively. Participants were medical students ranging from first to final year. The gender distribution was approximately balanced, and the average age was in the early 20s.

**TABLE 2:** Summary of study characteristics.

Study	Country	Study design	Participants (*n*)	Intervention	Teaching topic	Assessment of intervention	Kirkpatrick level
Achanta et al. [25]	UK	Qualitative	13	Online platform with 25 modules	ENT emergencies	Thematic analysis of semi‐structured interviews (perceptions)	2
Al‐Hussaini et al. [26]	UK	Qualitative	30	E‐book	ENT examinations	Thematic analysis of semi‐structured interviews (perceptions)	1
Alnabelsi et al. [27]	UK	RCT	25	Online lecture	ENT emergencies	SBA test on ENT emergencies (knowledge); Likert scale (satisfaction)	2
Chin et al. [28]	Australian	RCT	8	E‐learning (multimedia)	ENT examinations	OSCE checklist for ENT examinations (skill); Rating scale (satisfaction)	2
Dlugaiczyk et al. [29]	Germany	RCT	46	Mobile app (virtual patient)	BPPV treatment	Likert scale (satisfaction)	2
Dombrowski et al. [30]	Germany	Non‐RCT	109	Virtual learning environment online course	Core knowledge	Likert scale (satisfaction)	2
Edmond et al. [8]	UK	RCT	15	Podcast	Knowledge of epistaxis, otitis media, and tonsillitis	True/false MCQ on epistaxis, tonsillitis, and otitis media (knowledge); Likert (satisfaction)	2
Glicksman et al. [31]	Canada	RCT	23	Computer‐assisted instruction	Nasal packing technique	Time taken for nasal packing, and global rating scale for performance of procedure (skill)	2
Grasl et al. [32]	Austria	Non‐RCT	117	Web‐based learning ‘Unified Patient Program’	Case studies	SBA test on basic knowledge of ENT (knowledge); Likert scale (satisfaction)	2
Hu et al. [33]	Canada	Mixed‐methods	58	Web‐based platform	Anatomy of the larynx	Likert scale and free‐text (satisfaction)	2
Ijaz et al. [34]	UK	Pre‐and‐Post	75	Web‐based platform	ENT anatomy, common and emergency conditions, and clinical examinations	MCQ (knowledge); Likert scale (confidence)	2
Kandasamy et al. [35]	Canada	RCT	28	Computer‐assisted instruction	Pediatric stridor	MCQ (knowledge)	2
Kharidia et al. [36]	USA	Non‐RCT	38	Virtual conference course	ENT examinations	Likert scale for ENT examinations (confidence)	1
Kumar et al. [37]	India	Cross‐sectional survey	76	Online semester	ENT syllabus	Likert scale (attitude)	1
Lechner et al. [38]	Germany	Non‐RCT	31	Online course (podcast/video/cases)	ENT examinations	Self‐evaluation of examination skills with Likert scale (competence); OSCE for practical performance with global rating (skill)	2
Lee et al. [39]	Taiwan	RCT	30 in IM group; 30 in PPS group	Mobile gamified interactive multimedia	ENT emergent disorders	MCQ (knowledge); MST (competence); Global rating score (satisfaction)	2
Lyu et al. [52]	China	Case–Control	39	Online semester	ENT syllabus (theory only)	Likert scale (competence and satisfaction)	2
Michel et al. [40]	USA	Pre‐and‐post	365	Computer‐assisted instruction	High‐yield ENT topics	MCQ, case‐based, and clinical presentation formats (knowledge); Free‐text (satisfaction)	2
Mousseau et al. [41]	Canada	RCT	80	Multimedia module	Acute otitis media	Written test (knowledge); and VAS 100 mm scale (confidence); diagnostic accuracy compared to clinician's assessment (competence)	3
Pandya et al. [42]	UK	Cohort	16	Video linked lecture	Anatomy of the larynx	Likert scale and free‐text responses (attitude)	2
Pu et al. [43]	USA	Pre‐and‐post	147	Video	Tracheostomy care	MCQ (knowledge) and survey (confidence)	2
Samra et al. [44]	USA	RCT	7	Mobile app	Tympanic membrane pathology	Students' ability to interpret photographs of tympanic membranes (knowledge)	2
Shaira and Jayan [51]	India	Cross‐sectional survey	153	Virtual learning environment online course	ENT theory and practicals	Closed and open set, multiple choice, dichotomous questions, matrix rating, and Likert's scale (satisfaction)	1
Shetty et al. [45]	India	Cross‐sectional survey	170	Online classes	ENT curriculum	Survey on the opinion of online classes versus classroom learning; Agreement (yes/no) to questions about the experience of online classes	1
Steehler et al. [46]	USA	Mixed‐methods	5	Video conference course	ENT examination and common conditions	MCQ (knowledge); Likert survey and content analysis of free‐text responses (satisfaction)	2
Stepniak et al. [47]	Canada	RCT	21	Web‐based otoscopy simulator	Ear pathology	Short‐answer questions on videos of ear pathology (knowledge)	2
von Sass et al. [48]	Germany	Cross‐sectional survey	189	Web‐based case studies multimedia	Skull base	Likert scale (attitude)	2
Wu and Beyea [49]	Canada	RCT	18	Web‐based module	Ear pathology	Test to assess images of middle and external ear pathologies on ear simulator (knowledge); Clinical skills checklist for otoscopy examination (skill)	2
Wu et al. [50]	Canada	RCT	9	Web‐based module	Ear pathology	Checklist to evaluate otoscopy examination (skill); Test for correct identification of ear pathologies and normal ears on volunteer patients (knowledge); Survey with Likert scale and free‐text responses (confidence)	3

Abbreviations: MCQ, multiple choice question; MST, multimedia situational test; OSCE, objective structured clinical examination; SBA, single best answer; VAS, visual analogue scale.

**TABLE 3:** Summary of participant characteristics.

Authors	Year of study	Male	Female	Age
Achanta et al. [25]	3rd year	31%	69%	22 (median)
Al‐Hussaini et al. [26]	4th year	40%	60%	23 (median)
Alnabelsi et al. [27]	4th and 5th year	52%	48%	NR
Chin et al. [28]	Final year	NR	NR	NR
Dlugaiczyk et al. [29]	NR	NR	NR	NR
Dombrowski et al. [30]	5th year	NR	NR	NR
Edmond et al. [8]	2nd year	NR	NR	NR
Glicksman et al. [31]	1st year	48%	52%	NR
Grasl et al. [32]	Year not reported but at least completed 2 years	49%	51%	24.6 years (median), 23.8–25.6 (IQR)
Hu et al. [33]	1st year	38%	62%	23 years (mean); 1.8 years (SD)
Ijaz et al. [34]	Penultimate (37%) and final year (63%)	NR	NR	NR
Kandasamy et al. [35]	2nd year	NR	NR	NR
Kharidia et al. [36]	1st year	NR	NR	Median age 24.5
Kumar et al. [37]	Year not reported but stated sixth semester	NR	NR	NR
Lechner et al. [38]	4th and 5th year	26%	74%	25 (mean), 3.1 (SD)
Lee et al. [39]	NR	60%	40%	23 (median), 22–26 (range)
Lyu et al. [52]	6th year of 8‐year course	NR	NR	NR
Michel et al. [40]	3rd and 4th year	NR	NR	NR
Mousseau et al. [41]	3rd and 4th year	NR	NR	NR
Pandya et al. [42]	3rd year	NR	NR	NR
Pu et al. [43]	3rd year	NR	NR	NR
Samra et al. [44]	3rd year	NR	NR	NR
Shaira and Jayan [51]	Phase II and III	46%	54%	NR
Shetty et al. [45]	3rd year	46%	54%	20–24 (range)
Steehler et al. [46]	3rd and 4th year	58%	42%	NR
Stepniak et al. [47]	2nd year	NR	NR	NR
von Sass et al. [48]	Advanced medical students	42%	58%	NR
Wu & Beyea [49]	1st and 2nd year	NR	NR	NR
Wu et al. [50]	1st and 2nd year	NR	NR	NR

Abbreviations: IQR, interquartile range; NR, not reported; SD, standard deviation.

Among the 29 included studies, the majority were quantitative in design (*n* = 23, 79%), comprising RCTs (*n* = 12, 41%) [8, 27, 28, 29, 31, 35, 39, 41, 44, 47, 49, 50], non‐RCTs (*n* = 4, 14%) [30, 32, 36, 38], cross‐sectional surveys, case–control, and cohort studies (*n* = 6, 21%) [37, 42, 45, 48, 51, 52], and pre‐post studies (*n* = 3, 10%) [34, 40, 43]. Two studies (7%) were qualitative [25, 26], and two (7%) were mixed‐methods [33, 46]. The geographical spread of the studies was skewed toward high‐income countries, with the most frequent countries being Canada (*n* = 7, 24%), UK (*n* = 6, 21%), US (*n* = 5, 17%), and Germany (*n* = 4, 14%). Sample sizes varied widely, ranging from 5 to 365 participants.

E‐learning interventions included e‐books, mobile applications, podcasts, and multimedia modules. A broad range of ENT topics was covered, including anatomy, clinical examination, ENT emergencies, and ear pathology. The majority of studies (*n* = 22, 76%) assessed learning outcomes, corresponding to Kirkpatrick level 2. Five studies (17%) evaluated learner reactions (Level 1), while two studies (7%) assessed behavioral changes in clinical practice (Level 3).

### 3.3 Quality Assessment

All 29 studies were appraised using the MMAT tool—see Appendix S2. While the majority of RCTs demonstrated appropriate randomization procedures, limitations such as a lack of blinding and inconsistent monitoring of learner engagement may have introduced detection bias. Non‐randomized studies often mandated participation, which may have enhanced internal validity and generalizability. However, they were limited by inadequate control for confounders. The quantitative descriptive studies were generally well reported but relied on voluntary participation, thereby introducing potential non‐response bias. The qualitative studies demonstrated methodological rigor, typically employing thematic or content analysis. Findings were also supported by participant quotations. The limited integration of quantitative and qualitative data in mixed‐methods studies limited the depth of findings from these studies.

### 3.4 Quantitative Findings

#### 3.4.1 Knowledge

##### 3.4.1.1 Included Studies

Fourteen studies provided quantitative data on knowledge outcomes suitable for meta‐analysis [8, 27, 32, 34, 35, 39, 40, 41, 43, 44, 46, 47, 49, 50]. One study by Lee et al. [39] included two intervention arms with distinct e‐learning formats, and therefore was represented by two independent data points in our results.

##### 3.4.1.2 Pooled Effect Size and Heterogeneity

The initial pooled normalized gain in knowledge was 24.9% (95% C.I. 18.2–32.2). Heterogeneity was substantial, with *τ* ^2^ = 0.01 (95% C.I. 0.003–0.04), *I* ^2^ statistic was 75.2% (95% C.I. 42.8–88.5), and a significant *Q*‐test (*Q* = 56.5 (*p* < 0.0001)).

##### 3.4.1.3 Sensitivity Analysis

**FIGURE 3:**
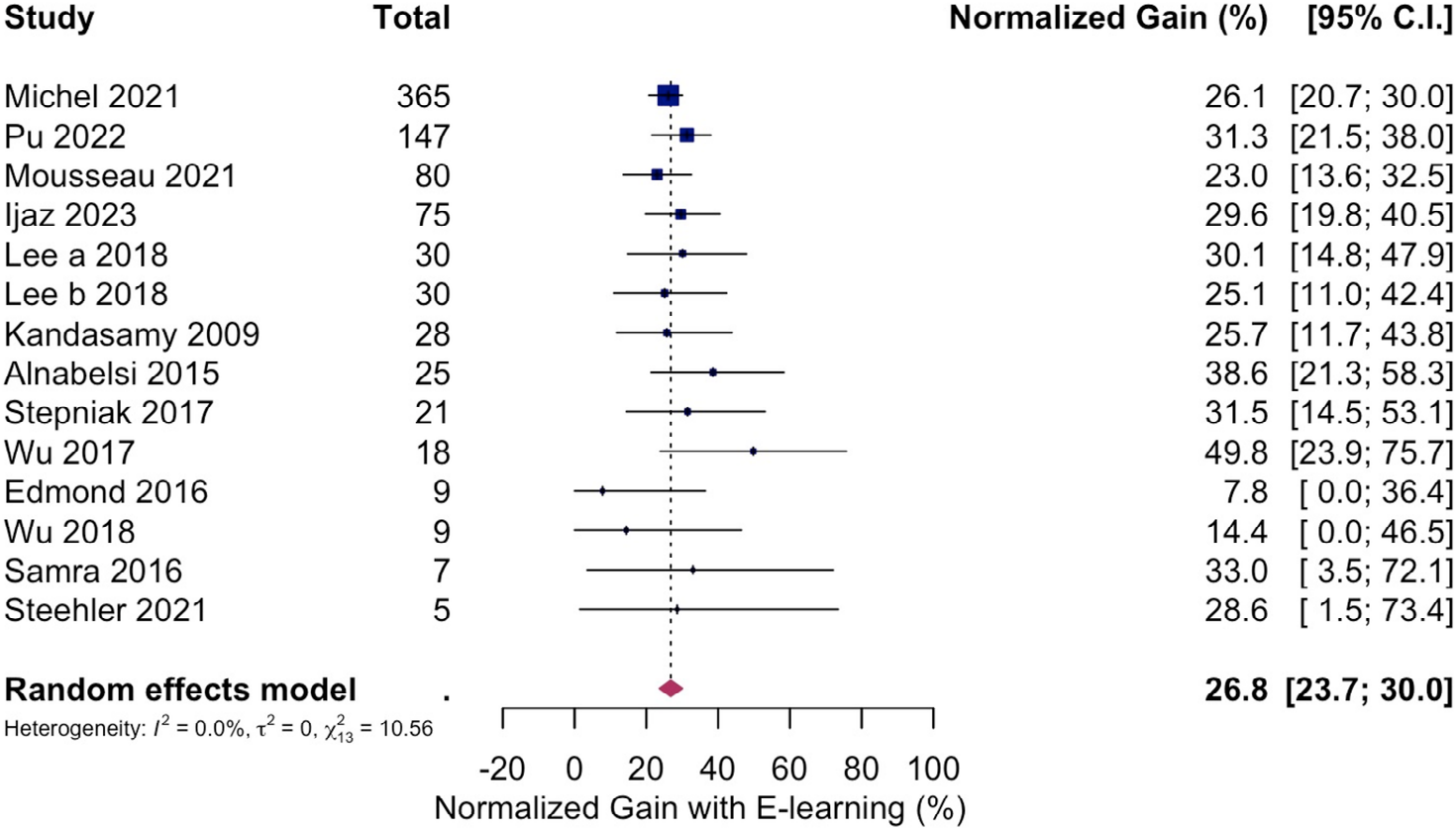
Forest plot for normalized gain in knowledge across 13 studies. [Color figure can be viewed in the online issue, which is available at www.laryngoscope.com]

A leave‐one‐out analysis identified Grasl et al. [32] as an influential outlier, reporting a low normalized gain of 4.6%. Excluding this study increased the pooled normalized gain to 26.8% (23.7%–30.0%), and reduced the heterogeneity (*I* ^2^ = 0%, 95% C.I. 0%–68.4%; *Q* = 10.6, *p* = 0.6; *τ* ^2^ = 0, 95% C.I. 0–0.01). As a result, this study was excluded from further analysis. The forest plot of the final pooled analysis is shown in Figure 3.

Wu & Beyea [49] reported a high normalized gain of 49.8% as seen on visual inspection of the forest plot (Figure 3). However, its exclusion had a negligible impact on the overall effect size (adjusted pooled normalized gain = 26.3%, 95% C.I. 23.2%–29.6%), hence it was not excluded. This study provided participants with repeated exposure to identical otoscopic images that were later used in the assessment, suggesting that repeated exposure may have contributed to high scores. Therefore, the repeat testing effect was investigated in the moderator analysis.

##### 3.4.1.4 Moderator Analyses

###### 3.4.1.4.1 Time Allocated to E‐Learning

The excluded study by Grasl et al. [32] reported that students in the e‐learning group spent approximately one‐third less time on the intervention compared with the lecture group. To explore whether time spent in e‐learning influenced knowledge gain, we analyzed data from 11 studies reporting allocated e‐learning time [8, 35, 39, 40, 41, 43, 44, 46, 47, 49, 50]. This ranged from 30 min to 8 weeks. Due to this wide variation, time was log‐transformed for analysis. The scatter plot (see Appendix S3) showed a flat regression line (slope coefficient −0.0005, *Z* (12) = −0.07, *p* = 0.9), with no statistically significant association (*QM* = 0.005, df = 1, *p* = 0.9).

###### 3.4.1.4.2 Synchronicity of E‐Learning Delivery

The lower knowledge gains in the e‐learning group reported by Grasl et al. [32] may potentially reflect differences in learner engagement. To investigate whether delivery format influenced outcomes, we performed a subgroup analysis comparing synchronous (real‐time) [27, 39, 41, 46, 49, 50] and asynchronous (self‐paced) e‐learning interventions [8, 34, 35, 40, 43, 44, 47].

Synchronized approaches yielded a pooled normalized gain of 27.5% (95% C.I. 21.1%–34.4%), compared to 26.7% (95% C.I. 23.2%–30.4%) for asynchronized formats. The forest plot is shown in Appendix S4. There was no statistically significant difference (*QM* = 0.10, df = 1, *p* = 0.8). An *I* ^2^ of 0% and *R* ^2^ of 0% suggested that the synchronicity of e‐learning delivery did not significantly influence normalized gain in knowledge.

###### 3.4.1.4.3 Repeat Testing Effect

Studies involving repeated assessments reported similar normalized gains (28.6%, 95% C.I. 22.7%–34.8%) [41, 43, 44, 46, 49], to those without (26.1%, 95% C.I. 22.5%–29.9%) [8, 27, 34, 35, 39, 40, 47, 50]. However, the difference was not statistically significant (*QM* = 0.8, df = 1, *p* = 0.4), and meta‐regression confirmed this (slope coefficient = 0.03, Z (12) = 0.9, *p* = 0.4), as shown in Appendix S5.

###### 3.4.1.4.4 Study Design

Subgroup analysis revealed similar normalized gains between RCTs (27.0%, 95% C.I. 21.4%–33.0%) [8, 27, 35, 39, 41, 44, 47, 49, 50] and pre‐post studies (26.8%, 95% C.I. 23.1%–30.7%) [34, 40, 43, 46], as shown in Appendix S6. Study design was not a significant moderator (*QM* = 0.01, df = 1, *p* = 0.9), supported by an *R* ^2^ of 0%.

###### 3.4.1.4.5 Student Level

Pooled normalized gains were comparable for early‐year students (28.2%, 95% C.I. 18.5–38.8) [8, 35, 47, 49, 50] and advanced‐year students (26.7%, 95% C.I. 23.1%–30.5%) [27, 34, 40, 41, 43, 44, 46], as shown in Appendix S7. The difference was not statistically significant (*QM* = 0.1, df = 1, *p* = 0.8), supported by an *R* ^2^ of 0%.

##### 3.4.1.5 Publication Bias

**FIGURE 4:**
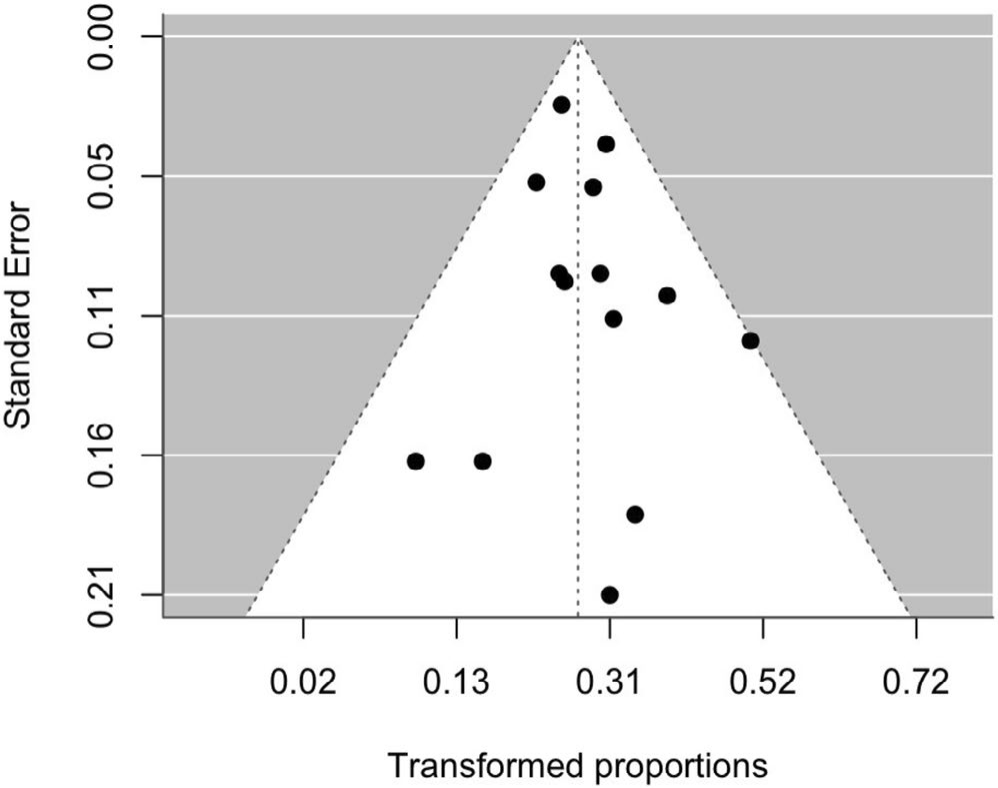
Funnel plot of study effect estimates on the x‐axis (double‐arcsine‐transformed proportion) for overall normalized gain against standard error as a measure of study size. The vertical line represents the value of the transformed summary effect, and the two limit lines indicate the 95% confidence intervals.

The funnel plot is displayed in Figure 4. Visual inspection showed a relatively symmetrical distribution of effect sizes, with most studies clustered around the pooled estimate within the 95% confidence limits. Egger's test detected no significant asymmetry (*z* = 0.6, *p* = 0.5), suggesting that publication bias was unlikely.

##### 3.4.1.6 E‐Learning Versus Traditional Teaching

Five studies compared knowledge gains between e‐learning and traditional teaching, comprising four RCTs [8, 27, 35, 41] and one quasi‐experimental study [32]. In the control arms, learners received face‐to‐face lectures [27, 32, 41] or written handouts [8, 35]; whereas the e‐learning interventions included online lectures, podcasts, web‐based modules, computer‐assisted instruction, and a multimedia module. Pre‐to‐post‐test improvements in the e‐learning groups ranged from 4.6% to 38.4%, compared with 8.8%–32.8% in the control groups.

As only Kandasamy et al. [35] reported standard deviations, a meta‐analysis of standardized mean differences (SMD) between e‐learning and control groups could not be performed. The direction of effect was mixed, with three studies reporting slightly higher gains in the e‐learning groups [27, 35, 41] while two marginally favored the control groups [8, 32].

### 3.5 Qualitative Findings

#### 3.5.1 Skill Development

Six studies assessed skill outcomes [28, 29, 38, 41, 49, 50], of which five were RCTs and one was a cohort study [38]. Skill domains included clinical examination skills [28, 38], performance of therapeutic maneuvers [29], and diagnostic interpretation of otoscopic images [41, 49, 50]. Assessment methods varied across studies, including checklists [28, 49, 50], written tests [29], rating scales [38], and diagnostic accuracy metrics [41].

Two studies found higher skill gains following e‐learning compared to traditional methods [28, 29]. For example, Chin et al. [28] reported mean exam scores of 78.5/99 for e‐learning versus 55.8/99 for traditional learning. Similarly, Dlugaiczyk et al. [29] observed higher pass rates with e‐learning (56.3%) than with traditional teaching (25.9%). Conversely, three studies [38, 41, 49] reported no significant differences between e‐learning and traditional methods in skill outcomes.

#### 3.5.2 Learner Confidence

Five studies evaluated changes in learner confidence [34, 36, 41, 43, 50], including two RCTs [41, 50], two pre‐post studies [34, 43], and one cohort study [36]. Confidence in knowledge [34, 43], skills [36, 41, 50], and diagnostic abilities [41, 50] was measured. Measurement tools differed, including five‐point Likert scales [34, 36, 50], a 10‐point rating scale [43], and a 100 mm visual analogue scale [41]. All studies reported an increase in self‐reported confidence following e‐learning interventions. Four studies demonstrated statistically significant improvements, while Mousseau et al. [41] observed a non‐significant improvement.

#### 3.5.3 Learner Perception and Experience

Twenty studies contributed qualitative or qualitized data suitable for thematic synthesis [8, 25, 26, 27, 29, 30, 31, 32, 33, 35, 37, 38, 39, 42, 43, 45, 46, 48, 51, 52]. Using line‐by‐line coding, a total of 84 unique codes were generated—see Appendix S8. These codes were consolidated into five descriptive themes, mapped in Appendix S9. From these, four overarching analytical themes emerged, reflecting key factors influencing the effectiveness of e‐learning in ENT education. These themes are listed in Table 4.

**TABLE 4:** Analytical themes.

1. Educational impact of e‐learning is maximized with multimedia elements.
2. Autonomy and control over e‐learning media empowers students to take charge of their education and foster self‐directed learning skills.
3. Technical issues undermine the adaptability and flexibility of the e‐learning experience.
4. E‐learning is a complementary tool rather than a stand‐alone solution, and is most effective alongside traditional face‐to‐face teaching.

### 3.6 Integrated Synthesis

The quantitative meta‐analysis demonstrated a pooled normalized knowledge gain of 26.8%. Knowledge improvement was observed consistently across diverse e‐learning modalities, and moderator analysis revealed no significant influence of study design, student level, delivery format (synchronous vs. asynchronous), duration of intervention, or repeat testing. These findings suggest that e‐learning was broadly effective regardless of contextual or methodological variations. Qualitative synthesis provided explanatory insights. Learners valued *multimedia‐rich content*, *flexibility*, and the *self‐directed nature* of e‐learning. These perceptions likely underpin the consistent knowledge gains that were observed in the quantitative analysis.

In contrast, findings related to *skills development* were mixed. Quantitative studies did not demonstrate a consistent advantage of e‐learning over traditional teaching methods. This aligned with qualitative reports that, while e‐learning facilitated theoretical understanding and procedural familiarization, it was *insufficient on its own for developing practical skills*. Students expressed a preference for *blended learning approaches*, combining digital tools with face‐to‐face clinical instruction.

Improvement in confidence was consistently observed in the quantitative studies. Several qualitative codes indicated that e‐learning facilitated self‐directed learning, helped identify knowledge gaps, and enhanced perceived preparedness and motivation, all of which aligned with the quantitative finding of improved learner confidence. However, both data sources highlighted factors that detracted from the overall learning experience, including the *impersonal nature* of e‐learning, lack of *real‐time feedback, and technical barriers*.

## 4 Discussion

### 4.1 Summary of Main Findings

This mixed‐methods systematic review synthesized current evidence on the effectiveness of e‐learning in undergraduate ENT education. Overall, e‐learning was found to improve knowledge acquisition, with a pooled normalized gain of 26.8%. Moderator analysis indicated that knowledge gain was not significantly influenced by student characteristics (learner level), intervention characteristics (synchronicity, duration, and presence of repeat testing), or study characteristics (study design). Knowledge gains were comparable in studies that compared e‐learning with traditional teaching, with only modest and inconsistent advantages for either modality. Confidence consistently improved, while skills development was more variable and, in some cases, inferior to traditional face‐to‐face teaching.

These findings align with the findings of McGann et al. [53], who similarly reported improvement in knowledge and confidence with online surgical skills training, but continued participant preference for in‐person teaching for skills. Similarly, Gormley et al. [54] found that while students felt e‐learning had a positive impact on clinical skills learning, they still considered hands‐on practice and face‐to‐face feedback as essential. These results suggest that while e‐learning is effective for building foundational knowledge and confidence, its capacity to develop clinical skills is limited unless combined with in‐person components. This reinforces one of the key findings from our integrated analysis, that students perceive e‐learning as a valuable adjunct rather than a replacement for face‐to‐face learning.

The effectiveness of e‐learning was enhanced by the inclusion of multimedia elements. This is consistent with Mayer's generative theory of multimedia learning [55] and Sweller's cognitive load theory [56], both of which emphasize the benefit of dual‐channel processing of verbal and pictorial information in improving retention. Additionally, learner autonomy and control were key contributors to the perceived success of e‐learning. This is consistent with studies that have shown interactive platforms that enable students to control the content, sequence, pace, and medium of instruction result in higher learner performance and satisfaction compared to less interactive platforms [57, 58, 59]. Learner autonomy aligns with the principle of learner‐centeredness, which prioritizes student agency, flexibility, and responsibility in the learning process in order to foster a deep learning experience [60]. Our findings, therefore, support the integration of e‐learning into ENT education to promote a learner‐centered environment.

### 4.2 Comparison With Existing Literature

Our findings align with those of Tarpada et al. [61], whose systematic review also concluded that e‐learning was effective in improving knowledge acquisition within ENT education. Additionally, they observed that nearly all included studies showed significant objective knowledge gains with e‐learning compared with traditional techniques. In contrast, our comparative analysis identified both studies in which e‐learning yielded greater gains and studies in which the control group performed better, with similar effect sizes. This pattern was mirrored in broader systematic reviews and meta‐analyses [62, 63, 64] across diverse health‐education settings, which reported either significantly higher gains with e‐learning, no significant differences, or mixed results. The evidence therefore suggests that e‐learning achieves knowledge outcomes comparable to traditional teaching.

The analysis by Tarpada et al. [61] was limited by substantial methodological heterogeneity, which prevented a quantitative synthesis. Consequently, their findings were presented narratively, without an estimation of pooled effect size. Other reviews on e‐learning in medical education have similarly relied on narrative syntheses, often encompassing multiple specialties and diverse healthcare professionals [63, 64, 65]. While these reviews contribute to the understanding of e‐learning in general, the heterogeneity in their study populations limits their applicability to a specific curricular area [66]. In contrast, our review focused exclusively on undergraduate ENT education and employed normalized gain as a common outcome metric, enabling a meta‐analysis that provided a precise and quantifiable estimate of e‐learning's effectiveness in improving knowledge. Our review may therefore generate relevant and applicable evidence for curriculum development.

### 4.3 Application of Our Findings

Medical students have reported higher satisfaction with web‐based learning tools compared to traditional resources [67], contributing to a surge in web‐based resources within ENT education [68]. This trend reflects a broader shift toward technology‐enhanced learning. While our review found e‐learning was associated with improved knowledge acquisition, the pooled normalized gain of 26.8% corresponded to a small effect size [18]. This may reflect limited alignment between assessments and learning content, and should be considered when interpreting the effectiveness of these interventions. Nonetheless, the relatively low gain highlights the importance of implementing e‐learning with clear pedagogical intent.

Rather than serving as a standalone solution, e‐learning should be integrated with traditional instructional methods, a view supported by previous research [57, 69, 70]. The flipped‐classroom model exemplifies this strategy, enabling learners to acquire foundational knowledge through e‐learning prior to attending face‐to‐face sessions in which the focus is on the attainment of higher‐order cognitive skills [71]. This approach is underpinned by adult learning principles, promoting active learning and self‐directed study [72]. When applied effectively, the flipped‐classroom model may therefore allow efficient use of limited curriculum time during ENT clinical placements.

The moderator analyses demonstrated that knowledge gain from e‐learning was not significantly associated with learner level, the synchronicity of delivery, or the duration of the intervention. This suggests that e‐learning can be effectively implemented across diverse learner populations, with flexibility in scheduling and duration. These findings support the adaptability of e‐learning as a teaching modality.

### 4.4 Strengths and Limitations

Conducting an SMD meta‐analysis comparing e‐learning with a control group was not feasible due to incomplete reporting. Among nine RCTs [8, 27, 35, 39, 41, 44, 47, 49, 50], two lacked standard deviations [8, 27], one did not report pre‐test scores [41], and another lacked a control group [39]. Instead, data from all nine RCTs, three pre‐post studies [34, 40, 43], one mixed‐methods study [46], and one non‐RCT [32], yielded a total of 14 studies with a complete pair of pre‐ and post‐test scores for e‐learning participants. A comparative meta‐analysis of these 14 data pairs was not possible as the pre‐ and post‐intervention groups were not independent, which is a prerequisite for an SMD statistical analysis [73]. To circumvent these limitations, we calculated normalized gain and employed a meta‐analysis of proportion. Normalized gain is increasingly utilized in medical education research to quantify learning outcomes [74, 75, 76, 77]. In this review, it enabled standardization of knowledge outcomes across studies, despite variation in data reporting. Meta‐analysis of proportion is also a recognized method for summarizing the effectiveness of educational interventions [78]. To our knowledge, this review is the first to apply normalized gain within a meta‐analytic framework in the medical education literature.

A key strength of this review lies in its mixed‐methods design, which enabled a comprehensive evaluation of the effectiveness of e‐learning by integrating quantitative effect estimates with qualitative insights. Thematic synthesis enriched our understanding of the impact of e‐learning by identifying learner‐reported features that contributed to the success of e‐learning, insights that would not have emerged from the quantitative data alone. Furthermore, the mixed‐methods approach maximizes the depth and applicability of review findings to inform real‐world practice [79].

Several limitations should be acknowledged. The exclusion of non‐English language studies and those published earlier than 15 years ago may have resulted in the omission of some relevant evidence. Reliance on pre‐ and post‐intervention data in our meta‐analysis may have introduced a potential repeat testing bias [80], although our moderator analysis showed no such effect was present. Methodological heterogeneity precluded a meta‐analysis of skill and confidence data.

We employed a ‘convergent segregated’ approach to synthesis and integration, as recommended in the JBI methodological guidance [17], because the quantitative studies (knowledge gain) and qualitative studies (student experiences) addressed different dimensions of e‐learning in ENT. This preserved the methodological rigor of each stream before drawing meta‐inferences. However, because integration occurred only at the level of synthesized findings rather than individual studies, the depth of cross‐data interaction may have been reduced. An alternative approach within a segregated framework is the Bayesian mixed‐methods synthesis [81], which transforms both data types into a common format and uses Bayesian modeling to produce integrated probabilistic estimates that incorporate uncertainty from both sources. While methodologically robust, this approach can introduce bias through subjective coding of qualitative data and is resource‐intensive, requiring specialist statistical expertise [81].

The *overall methodological quality* of the included studies, as assessed by the MMAT, was deemed *moderate*. Common limitations included *incomplete control for confounding* in non‐randomized studies, *lack of blinding* of outcome assessors in RCTs, and *limited integration* in mixed‐methods designs. These factors highlight the need for more rigorously designed studies.

### 4.5 Future Research Directives

Mayer [82] described a robust framework for optimizing multimedia design, aimed at reducing extraneous cognitive load and enhancing learner engagement. Future research should focus on evaluating such design features to determine the optimal conditions that most effectively improve learning outcomes. This requires rigorous study designs such as well‐powered RCTs.

There is also a pressing need for more *qualitative and mixed‐methods studies* to explore *learners' perspectives* and experiences of different e‐learning design features. Such research would help inform the development of e‐learning strategies that *align with learners' preferences and needs*.

## 5 Conclusion

E‐learning enhances knowledge acquisition and learner confidence in undergraduate ENT education, supported by multimedia elements, learner autonomy, and flexible delivery. However, its effectiveness as a standalone tool is limited, particularly in skill development, necessitating a blended learning approach.

Future research should be aimed at refining e‐learning design by addressing technical barriers and applying evidence‐based multimedia principles. By optimizing its design and strategic use, e‐learning can serve as a valuable and complementary component to undergraduate ENT education.

## Conflicts of Interest

The authors declare no conflicts of interest.

## Supporting information


**Appendix S1:** Database searches.

**Appendix S2:** MMAT findings.

**Appendix S3:** Scatter plot for time allocated to e‐learning (*n* = 12) with regression line and corresponding 95% confidence interval boundaries.

**Appendix S4:** Subgroup analysis for synchronized interventions (*n* = 7) versus asynchronized interventions (*n* = 7).

**Appendix S5:** Scatter plot for repeat testing (*n* = 14) with regression line and corresponding 95% confidence interval boundaries.

**Appendix S6:** Subgroup analysis of study design with RCT (*n* = 10) versus pre‐post studies (*n* = 4).

**Appendix S7:** Subgroup analysis of the effect of early‐ (*n* = 6) and advanced‐year (*n* = 7) medical students on normalized gain.

**Appendix S8:** Line‐by‐line coding stage of thematic synthesis.

**Appendix S9:** Mapping matrix of studies to descriptive themes.

## Data Availability

The data that support the findings of this study are available from the corresponding author upon reasonable request.
