# Supplementary material for: Effectiveness of E‐Learning in Undergraduate ENT Education: A Mixed‐Methods Systematic Review
Source: Laryngoscope. 2025 Sep 27;136(3):1062–76. doi: 10.1002/lary.70164 (PMC12913759; doi:10.1002/lary.70164)
Supplement: Supplementary file 1 — Appendix S1: Database searches. [file LARY-136-1062-s005.docx]

*Supplementary File I: Database searches*

| Database | Search query |
| --- | --- |
| Ovid MEDLINE (1946 to February Week 2 2025) | 1. Otolaryngology/ or ENT.mp or Otorhinolaryngologic Diseases/  2. “Ear Nose and Throat”.mp.  3. Otorhinolaryngology.mp.  4. 1 or 2 or 3  5. Computer-Assisted Instruction/ or Education, Distance/ or “Electronic learning”.mp or Internet/  6. E-Learning.mp.  7. Web-based.mp.  8. Digital.mp.  9. Online.mp.  10. Virtual.mp.  11. 5 or 6 or 7 or 8 or 9 or 10  12. Students, Medical/ or Medical Student*.mp. or Education, Medical, Undergraduate/  13. Undergraduate.mp.  14. Medical Course.mp.  15. Medical School*.mp. or Schools, Medical/  16. Universities/ or Universit*.mp.  17. 12 or 13 or 14 or 15 or 16  18. 4 and 11 and 17  19. limit 18 to (English language and last 20 years) |
| Ovid Embase (1974 to 2025 Week 28) | 1. ENT.mp. or otorhinolaryngology/  2. ear nose throat disease/ “Ear Nose and Throat”.mp. or otorhinolaryngology/  3. Otolaryngology.mp.  4. 1 or 2 or 3  5. “Electronic learning”.mp. or e-learning/  6. Internet/ or e-learning/  7. “Computer Assisted Instruction”.mp  8. Internet/ or Web-based.mp.  9. Online.mp.  10. Digital.mp.  11. Virtual.mp.  12. Distance.mp.  13. 5 or 6 or 7 or 8 or 9 or 10 or 11 or 12  14. medical student/ or Medical Student*.mp.  15. Undergraduate.mp.  16. Medical Course.mp.  17. medical school/ or Medical School*.mp.  18. Universit*.mp. or university/  19. 14 or 15 or 16 or 17 or 18  20. 4 and 13 and 19  21. limit 20 to (English language and last 20 years) |
| EBSCO Education Research Complete | S1. Medical Student* OR Undergraduate OR Medical Course OR Medical School* OR Universi*  S2. “Electronic learning” OR E-learning OR “Computer Assisted Instruction” OR Web-based OR Online OR Digital OR Virtual OR Distance OR Internet  S3. “ENT” OR (“Ear Nose and Throat”) OR Otolaryngology OR Otorhinolaryngology  S4. S1 AND S2 AND S3 |
| Web of Science Core Collection (1900 – present) | (TS=(ENT) OR TS=(“Ear Nose and Throat”) OR TS=(Otolaryngology) OR TS=(Otorhinolaryngology)) AND (TS=(“Electronic learning”) OR TS=(E-learning) OR TS=(“Computer Assisted Instruction”) OR TS=(Web-based) OR TS=(Online) OR TS=(Digital) OR TS=(Virtual) OR TS=(Distance) OR TS=(Internet)) AND (TS=(Medical Student*) OR TS=(Undergraduate) OR TS=(Medical Course) OR TS=(Medical School*) OR TS=(Universit*)) and English (Languages) |
