# Supplementary material for: Effectiveness of E‐Learning in Undergraduate ENT Education: A Mixed‐Methods Systematic Review
Source: Laryngoscope. 2025 Sep 27;136(3):1062–76. doi: 10.1002/lary.70164 (PMC12913759; doi:10.1002/lary.70164)
Supplement: Supplementary file 2 — Appendix S2: MMAT findings. [file LARY-136-1062-s001.docx]

*Supplementary File 2: MMAT findings.*

| Quantitative randomized controlled trials | | | | | | |
| --- | --- | --- | --- | --- | --- | --- |
|  | Study | 1. Is randomization appropriately performed? | 2. Are the groups comparable at baseline? | 3. Are there complete outcome data? | 4. Are outcome assessors blinded? | 5. Did the participants adhere to the assigned intervention? |
| 1 | Alnabelsi et al (2015) | 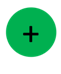 | 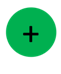 | 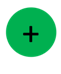 | 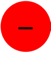 | 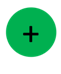 |
| 2 | Chin et al (2019) | 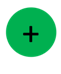 | 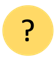 | 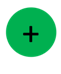 | 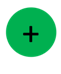 | 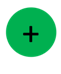 |
| 3 | Dlugaiczyk et al (2018) | 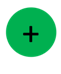 | 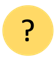 | 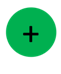 | 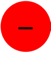 | 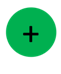 |
| 4 | Edmond et al (2016) | 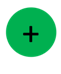 | 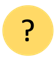 | 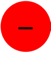 | 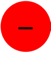 | 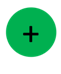 |
| 5 | Glicksman (2009) | 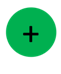 | 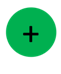 | 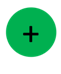 | 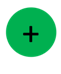 | 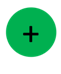 |
| 6 | Kandasamy (2009) | 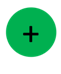 | 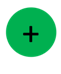 | 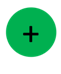 | 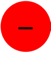 | 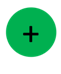 |
| 7 | Lee et al (2018) | 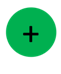 | 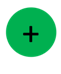 | 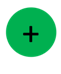 | 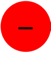 | 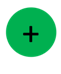 |
| 8 | Mousseau et al (2021) | 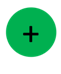 | 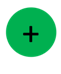 | 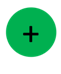 | 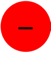 | 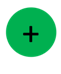 |
| 9 | Samra et al (2016) | 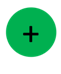 | 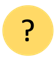 | 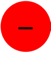 | 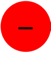 | 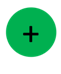 |
| 10 | Stepniak (2017) | 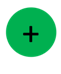 | 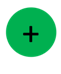 | 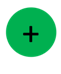 | 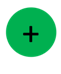 | 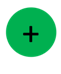 |
| 11 | Wu & Beyea | 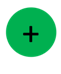 | 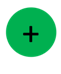 | 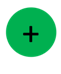 | 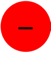 | 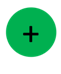 |
| 12 | Wu et al (2018) | 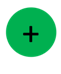 | 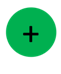 | 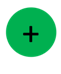 | 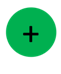 | 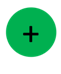 |
| 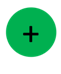= Yes 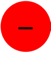 = No 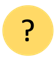 = Can’t tell | | | | | | |

| Quantitative non-randomized studies | | | | | | |
| --- | --- | --- | --- | --- | --- | --- |
|  | Study | 1. Are the participants representative of the target population? | 2. Are measurements appropriate regarding both the outcome and intervention? | 3. Are there complete outcome data? | 4. Are the confounders accounted for in the design and analysis? | 5. During the study period, is the intervention administered as intended? |
| 1 | Dombrowski et al (2018) | 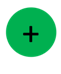 | 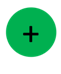 | 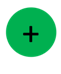 | 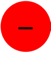 | 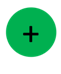 |
| 2 | Grasl et al (2012) | 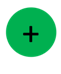 | 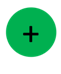 | 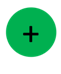 | 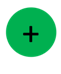 | 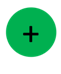 |
| 3 | Kharidia et al (2023) | 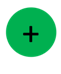 | 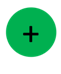 | 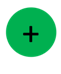 | 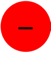 | 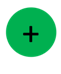 |
| 4 | Lechner et al (2022) | 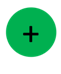 | 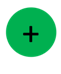 | 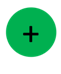 | 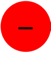 | 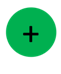 |
| 5 | Lyu et al (2024) | 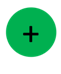 | 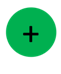 | 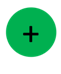 | 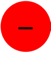 | 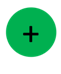 |
| 6 | Pandya et al (2021) | 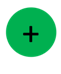 | 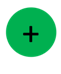 | 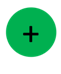 | 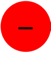 | 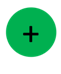 |
| 7 | Ijaz et al (2023) | 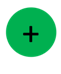 | 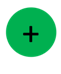 | 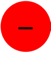 | 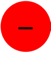 | 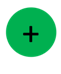 |
| 8 | Michel et al (2021) | 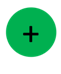 | 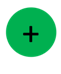 |  |  |  |
| 9 | Pu et al (2022) |  |  |  |  |  |
| 10 | Steehler et al (2021) |  |  |  |  |  |
| = Yes = No = Can’t tell | | | | | | |

| Quantitative descriptive studies | | | | | | |
| --- | --- | --- | --- | --- | --- | --- |
|  | Study | 1. Is the sampling strategy relevant? | 2. Is the sample representative of the target population? | 3. Are the measurements appropriate? | 4. Is the risk of nonresponse bias low? | 5. Is the statistical analysis appropriate? |
| 1 | Hu et al (2009) |  |  |  |  |  |
| 2 | Kumar et al (2023) |  |  |  |  |  |
| 3 | Shaira & Jayan (2024) |  |  |  |  |  |
| 4 | Shetty et al (2022) |  |  |  |  |  |
| 5 | von Sass et al (2015) |  |  |  |  |  |
| = Yes = No = Can’t tell | | | | | | |

| Mixed-methods studies | | | | | | |
| --- | --- | --- | --- | --- | --- | --- |
|  | Study | 1. Is there an adequate rationale for using a mixed methods design? | 2. Are the different components of the study effectively integrated? | 3. Are the outputs of the integration adequately interpreted? | 4. Are divergences and inconsistencies adequately addressed? | 5. Do the different components adhere to the quality criteria of each tradition of the methods involved? |
| 1 | Hu et al (2009) |  |  |  |  |  |
| 2 | Steehler et al (2021) |  |  |  |  |  |
| = Yes = No = Can’t tell | | | | | | |

| Qualitative studies | | | | | | |
| --- | --- | --- | --- | --- | --- | --- |
|  | Study | 1. Is the qualitative approach appropriate to answer the research question? | 2. Are the qualitative data collection methods adequate? | 3. Are the findings adequately derived from the data? | 4. Is the interpretation of results sufficiently substantiated by data? | 5. Is there coherence between qualitative data sources, collection, analysis and interpretation? |
| 1 | Achanta et al (2023) |  |  |  |  |  |
| 2 | Al-Hussaini et al (2016) |  |  |  |  |  |
| 3 | Hu et al (2009) |  |  |  |  |  |
| 4 | Steehler et al (2021) |  |  |  |  |  |
| = Yes = No = Can’t tell | | | | | | |
