# Supplementary figures and images for: Effectiveness of E‐Learning in Undergraduate ENT Education: A Mixed‐Methods Systematic Review
Source: Laryngoscope. 2025 Sep 27;136(3):1062–76. doi: 10.1002/lary.70164 (PMC12913759; doi:10.1002/lary.70164)

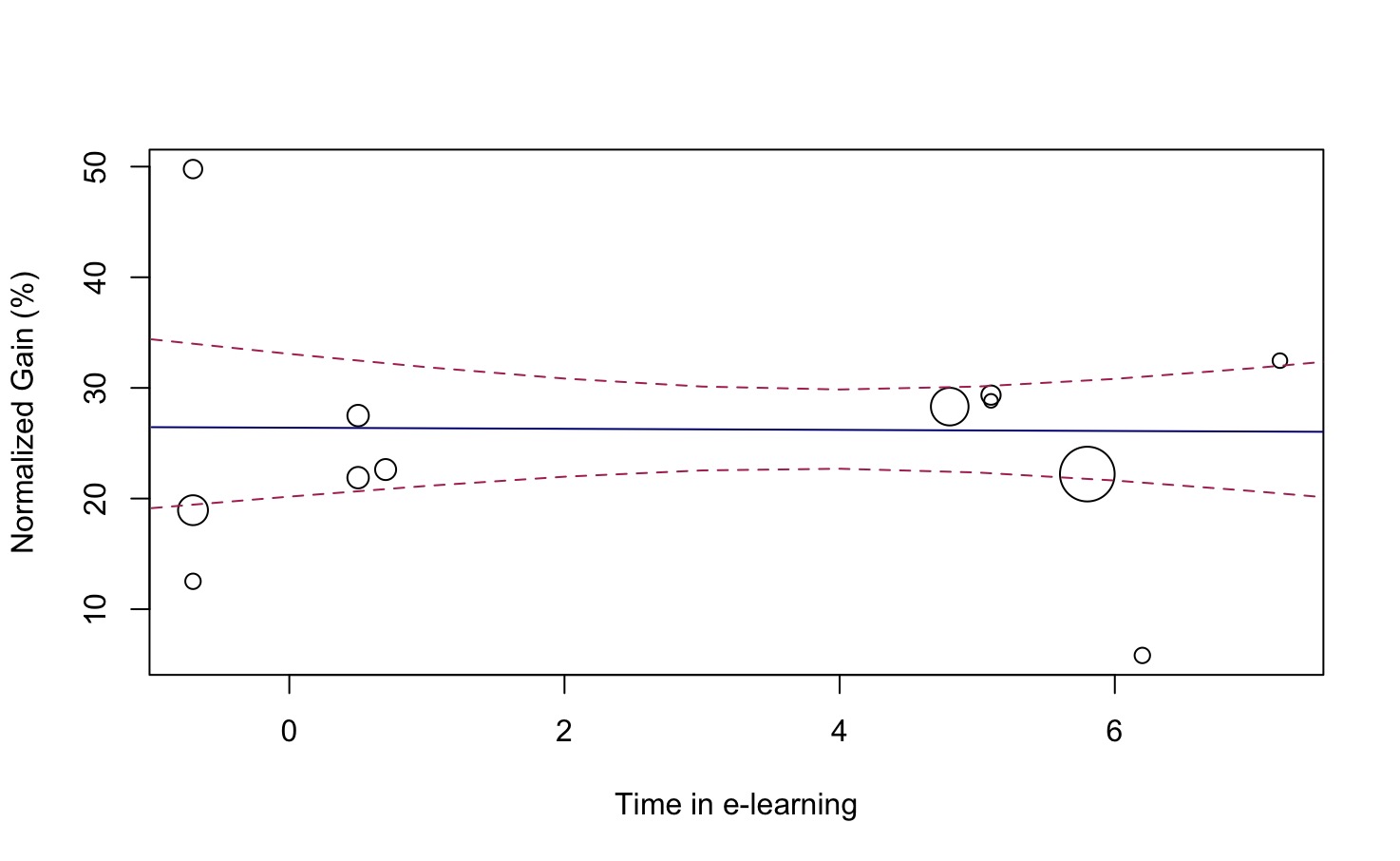

Supplement: Supplementary file 3 — Appendix S3: Scatter plot for time allocated to e‐learning (n = 12) with regression line and corresponding 95% confidence interval boundaries. [file LARY-136-1062-s004.jpeg]

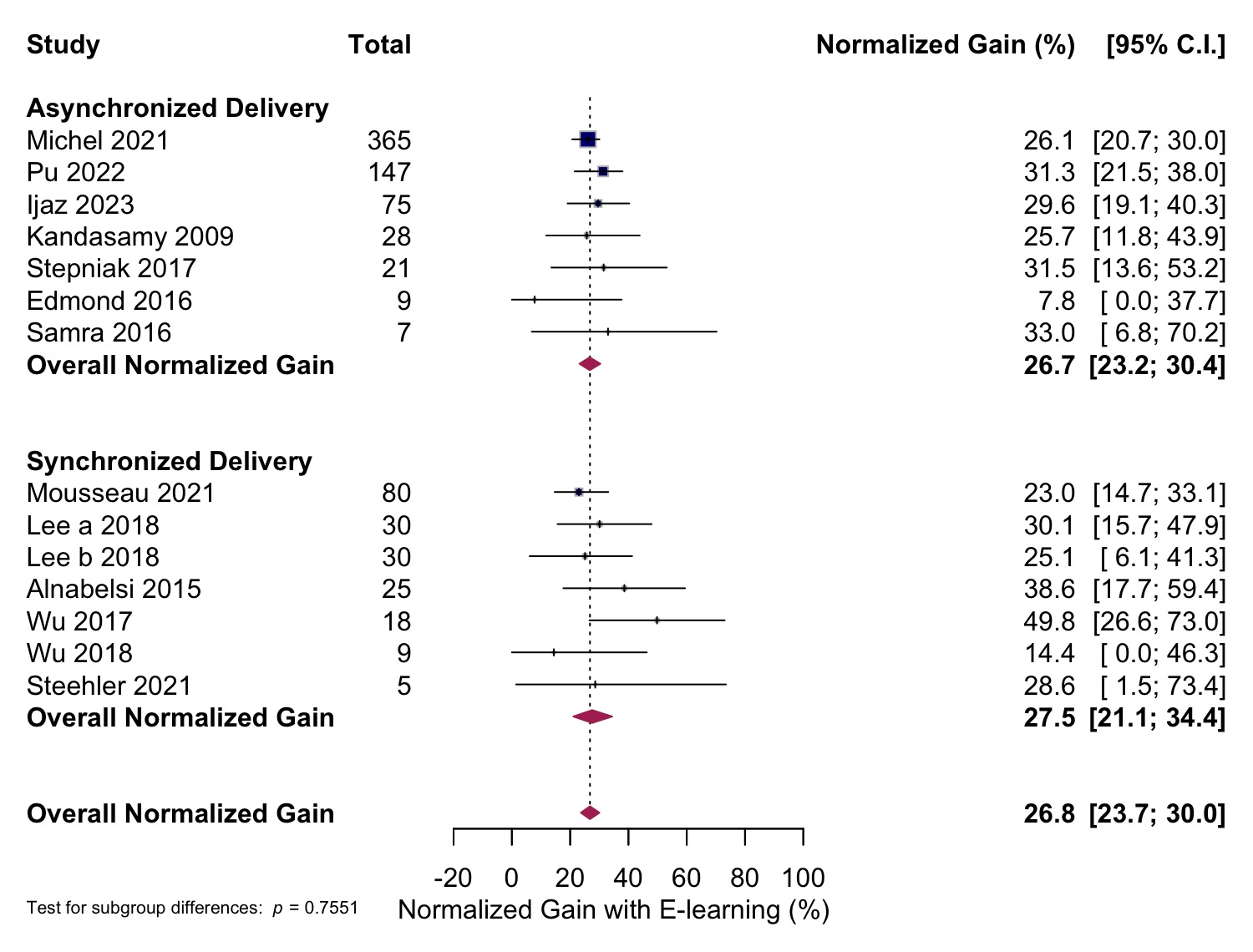

Supplement: Supplementary file 4 — Appendix S4: Subgroup analysis for synchronized interventions (n = 7) versus asynchronized interventions (n = 7). [file LARY-136-1062-s007.jpeg]

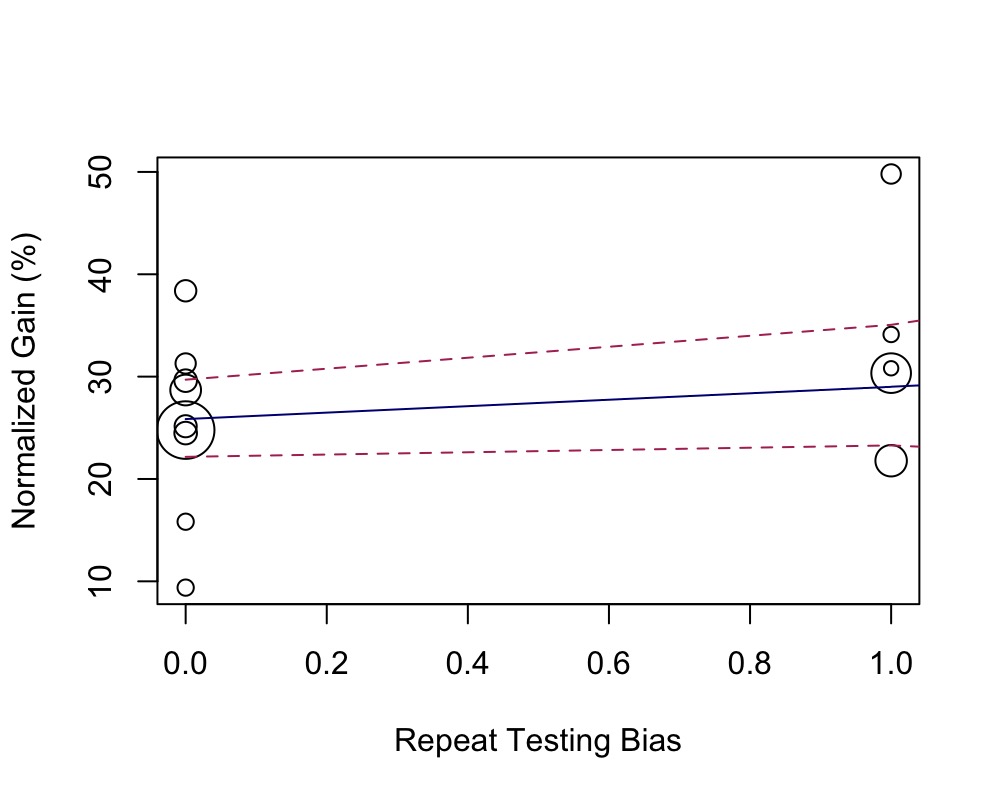

Supplement: Supplementary file 5 — Appendix S5: Scatter plot for repeat testing (n = 14) with regression line and corresponding 95% confidence interval boundaries. [file LARY-136-1062-s006.jpeg]

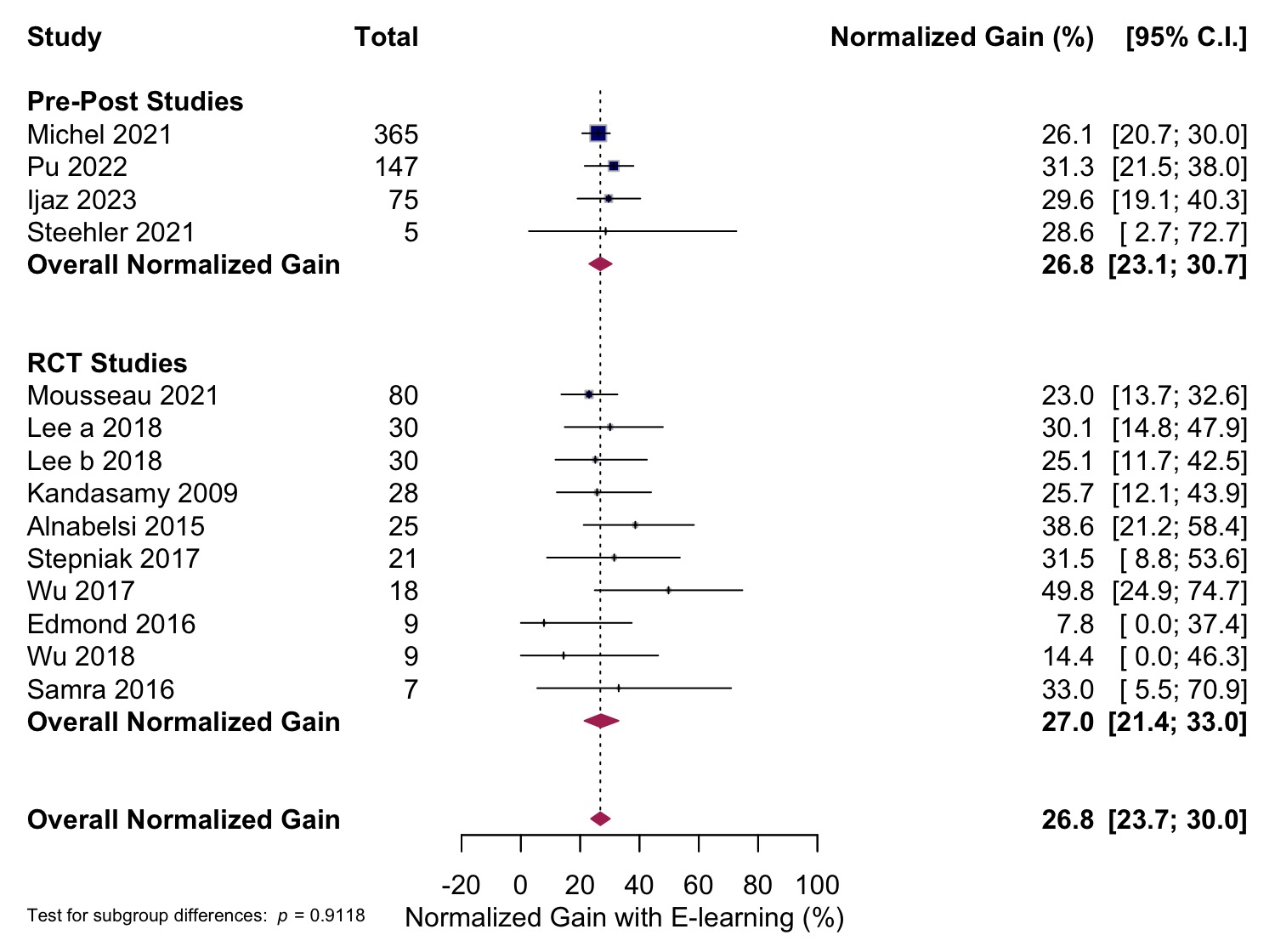

Supplement: Supplementary file 6 — Appendix S6: Subgroup analysis of study design with RCT (n = 10) versus pre‐post studies (n = 4). [file LARY-136-1062-s009.jpeg]

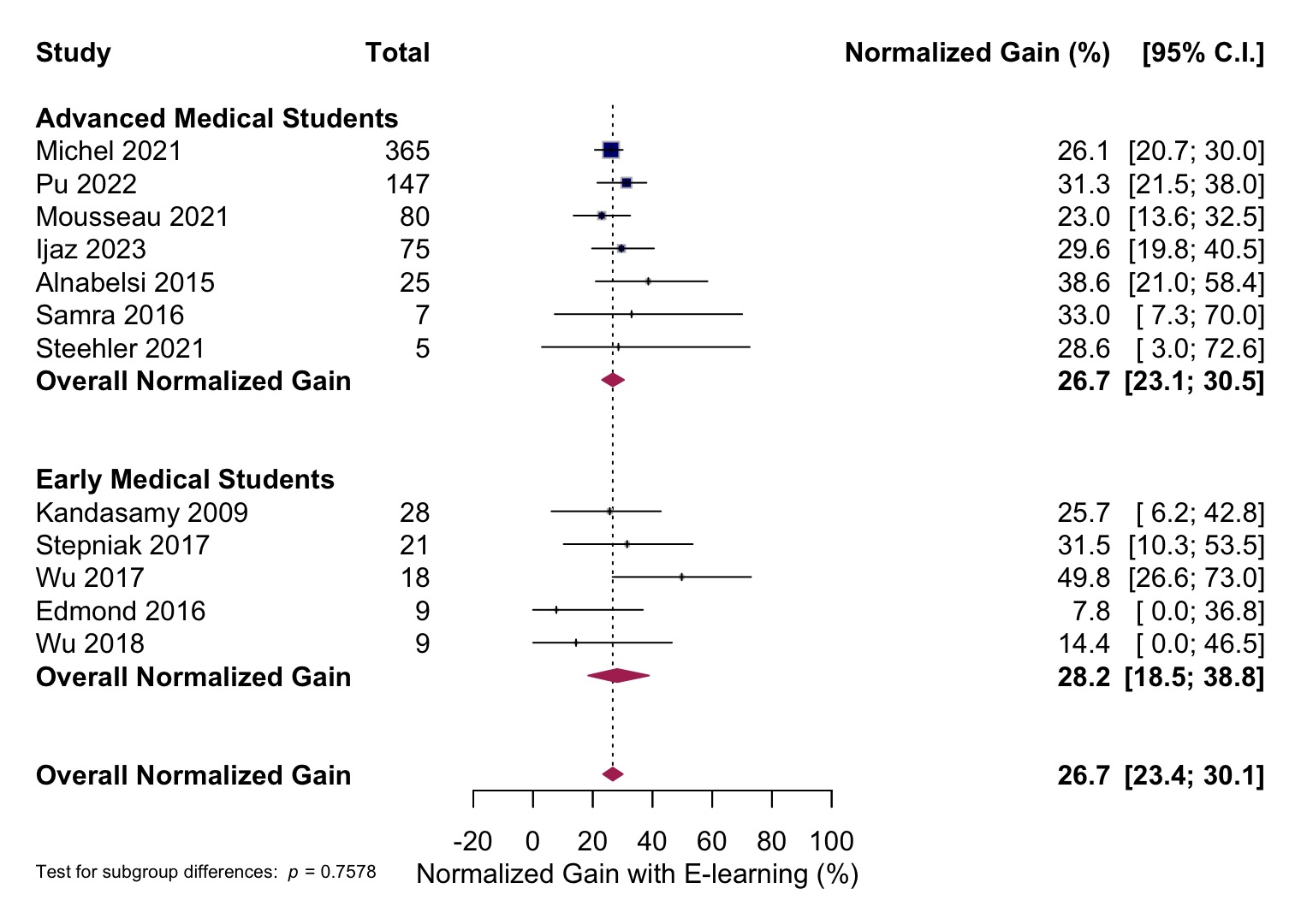

Supplement: Supplementary file 7 — Appendix S7: Subgroup analysis of the effect of early‐ (n = 6) and advanced‐year (n = 7) medical students on normalized gain. [file LARY-136-1062-s008.jpeg]
