# Supplementary material for: Effectiveness of E‐Learning in Undergraduate ENT Education: A Mixed‐Methods Systematic Review
Source: Laryngoscope. 2025 Sep 27;136(3):1062–76. doi: 10.1002/lary.70164 (PMC12913759; doi:10.1002/lary.70164)
Supplement: Supplementary file 8 — Appendix S8: Line‐by‐line coding stage of thematic synthesis. [file LARY-136-1062-s002.docx]

*Supplementary File 7:* *Line-by-line coding stage of thematic synthesis.*

| Study | Codes |
| --- | --- |
| Achanta et al (2023) | - Lack of immediate feedback - More easily distracted during e-learning - Inefficient note taking opportunities during e-learning - Caters to different learning styles - Interactivity element facilitated engagement - Flexible, accessible, and convenient - Ability to control and review content material - Desire for procedural videos |
| Al-Hussaini et al (2016) | - Clear and user-friendly presentation - Desire for more interactivity - High educational value of multimedia integration - Need for clinical examination videos - Effective self-study tool for consolidating learning |
| Alnabelsi et al (2015) | - Intermittent audio–visual problems - High perceived usefulness - Moderate satisfaction with interactivity - Highly meets educational needs - High overall satisfaction but slightly higher with face-to-face teaching |
| Dlugaiczyk et al (2018) | - Superior media quality in delivering content than traditional teaching - Comparable high learning success in both formats |
| Dombrowski et al (2018) | - High satisfaction with quality and usability - Leads to better preparation for the practical course - Knowledge gains - Support for further development of e-learning |
| Edmond et al (2016) | - Strong endorsement of video podcasts in medical education - Usefulness of video podcasts compared to traditional handouts varied from neutral opinion to agreement. - Valued as a learning resource - More enjoyable than traditional handouts |
| Glicksman et al (2009) | - Clear preference for computer-assisted learning over traditional text-based methods |
| Grasl et al (2012) | - Slightly higher satisfaction with traditional lecturing compared to web-based learning - Web-based learning program boosts motivation - Web-based learning program moderately improves their relation to practical experience - Increases perceived effort in terms of time and costs |
| Hu et al (2009) | - Appreciation for 3D visualization in understanding anatomy - User-friendly interface - Preference for 3D models to supplement lectures - Mixed enjoyment with 3D computer module - Reduced motivation due to technical problems with access - Suboptimal pace of instructional material - Information overload |
| Kandasamy et al (2009) | - Preference for computer-assisted learning over online articles |
| Kumar et al (2023) | - Helpful in understanding basic ENT concepts - Online classes supplement traditional face-to-face methods - Mixed perceptions of the value of e-learning in clinical skills - Inferiority of e-learning to teach examination techniques compared to physical examination - Teachers had adequate technical knowledge for online classes |
| Lechner et al (2022) | - Online training cannot replace practical teaching - Neutral opinion on gaining knowledge for practical ENT skills - Neutral opinion on preparation for clinical exams - Online courses provide adequate preparation for subsequent in-class teaching - Motivator for further skills development |
| Lee et al (2018) | - High satisfaction with mobile technology - Greater satisfaction with interactive multimedia over PowerPoint video lectures |
| Lyu et al (2024) | - High self-perceived mastery of knowledge of anatomy and clinical conditions |
| Pu et al (2022) | - High perceived helpfulness of video-based learning |
| Pandya et al (2021) | - Moderate improvement in anatomical knowledge but perceived as inferior to physical model - Mixed satisfaction compared to face-to-face teaching - Desire for more practical and clinical examples - Encouragement to learn ENT - Clare and relevant content - Visual aids enhanced understand - Concise and easy to understand - Audio-visual problems - Impersonal method of teaching - Mixed feelings about pacing of videos |
| Shaira & Jayan (2024) | - Students appreciated flexible, repeatable content, particularly short videos - Students missed in-person clinical experience and bedside teaching - Challenges included eye strain, neck pain, home distractions, and procrastination, and maintaining motivation and focus - Benefits included being with family, saving money, and flexible time management |
| Shetty et al (2022) | - Convenience of online learning - Lack of social and practical skills - Connectivity challenges with online classes - Health and wellbeing concerns with prolonged screen time - Preference for a blended learning approach with online and classroom teaching |
| Steehler et al (2021) | - Technical issues with video streaming - Desire for more surgical videos - Students enjoyed surgical videos - Concerns about lengthy videos |
| von Sass et al (2015) | - Enhanced analytical and clinical problem-solving skills - Suitability of interactive multimedia for complex topics - Motivated by the relevance of e-learning content to final examinations - Demand for more in-class lectures - Preference for optional e-learning for self-directed hybrid learning - E-learning helped identify and address knowledge gaps |
