# Supplementary material for: Effectiveness of E‐Learning in Undergraduate ENT Education: A Mixed‐Methods Systematic Review
Source: Laryngoscope. 2025 Sep 27;136(3):1062–76. doi: 10.1002/lary.70164 (PMC12913759; doi:10.1002/lary.70164)
Supplement: Supplementary file 9 — Appendix S9: Mapping matrix of studies to descriptive themes. [file LARY-136-1062-s003.docx]

*Supplementary File 8:* *Mapping matrix of studies to descriptive themes*

| Descriptive themes | | | | | |
| --- | --- | --- | --- | --- | --- |
| Study | 1. Valued educational experience | 2. Enhanced engagement and motivation with multimedia | 3. Technical difficulties and impersonal | 4. Preference for blended learning approaches | 5. High adaptability and flexibility |
| Achanta et al (2023) |  | ✓ | ✓ |  | ✓ |
| Al-Hussaini et al (2016) | ✓ | ✓ |  |  |  |
| Alnabelsi et al (2015) | ✓ |  | ✓ | ✓ |  |
| Dlugaiczyk et al (2018) | ✓ |  |  | ✓ |  |
| Dombrowski et al (2018) | ✓ |  |  | ✓ |  |
| Edmond et al (2016) | ✓ | ✓ |  |  |  |
| Glicksman et al (2009) | ✓ |  |  |  |  |
| Grasl et al (2012) |  | ✓ |  | ✓ |  |
| Hu et al (2009) | ✓ | ✓ | ✓ | ✓ |  |
| Kandasamy et al (2009) | ✓ |  |  |  |  |
| Kumar et al (2023) | ✓ |  |  | ✓ |  |
| Lechner et al (2022) |  |  |  | ✓ |  |
| Lee et al (2018) | ✓ | ✓ |  |  |  |
| Lyu et al (2024) | ✓ |  |  |  |  |
| Pu et al (2022) | ✓ |  |  |  |  |
| Pandya et al (2021) |  |  | ✓ | ✓ |  |
| Shaira & Jayan (2024) |  |  | ✓ | ✓ | ✓ |
| Shetty et al (2022) |  |  | ✓ | ✓ | ✓ |
| Steehler et al (2021) | ✓ |  | ✓ |  |  |
| von Sass et al (2015) | ✓ | ✓ |  | ✓ | ✓ |
